# Sex-Specific Impacts of Exercise on Cardiovascular Remodeling

**DOI:** 10.3390/jcm10173833

**Published:** 2021-08-26

**Authors:** Rifat A. Islam, Siri Sham S. Khalsa, Arpita K. Vyas, Roshanak Rahimian

**Affiliations:** 1 Department of Physiology and Pharmacology, Thomas J. Long School of Pharmacy, University of the Pacific, Stockton, CA 95211, USA; r_islam2@u.pacific.edu; 2 College of Medicine, California Northstate University, Elk Grove, CA 95757, USA; SiriShamSunder.Khalsa6641@cnsu.edu

**Keywords:** sex differences, exercise, cardiac remodeling, vascular remodeling, exercise-induced cardiovascular remodeling (EICR)

## Abstract

Cardiovascular diseases (CVD) remain the leading cause of death in men and women. Biological sex plays a major role in cardiovascular physiology and pathological cardiovascular remodeling. Traditionally, pathological remodeling of cardiovascular system refers to the molecular, cellular, and morphological changes that result from insults, such as myocardial infarction or hypertension. Regular exercise training is known to induce physiological cardiovascular remodeling and beneficial functional adaptation of the cardiovascular apparatus. However, impact of exercise-induced cardiovascular remodeling and functional adaptation varies between males and females. This review aims to compare and contrast sex-specific manifestations of exercise-induced cardiovascular remodeling and functional adaptation. Specifically, we review (1) sex disparities in cardiovascular function, (2) influence of biological sex on exercise-induced cardiovascular remodeling and functional adaptation, and (3) sex-specific impacts of various types, intensities, and durations of exercise training on cardiovascular apparatus. The review highlights both animal and human studies in order to give an all-encompassing view of the exercise-induced sex differences in cardiovascular system and addresses the gaps in knowledge in the field.

## 1. Introduction

A topic of great interest over the past two decades has been the impact of exercise on the cardiovascular structure and function [1,2]. Exercise-induced cardiovascular remodeling (EICR) is often a result of adaptive, structural, functional, and molecular changes caused by exercise training. Although the term EICR is more recent, its study dates originated to over a century ago when researchers noted enlarged hearts in Nordic skiers and American university rowers [3,4]. Since then, several forms of EICR have been reported. Documented forms of EICR include chamber dilation, ventricular hypertrophy, increased coronary reserve [5], and enhanced diastolic filling [6]. Unlike the adverse outcomes of pathological cardiovascular remodeling, EICR often results in improved cardiovascular functions, including increased cardiac output and decreased resting heart rate. Furthermore, EICR has been shown to lower the risk of cardiovascular diseases (CVD), one of the leading causes of death worldwide [7], such as arrhythmias, heart failure, and ischemic conditions [8,9].

Another emerging area less well studied in cardiovascular remodeling is the impact of sex-specific exercise-induced alteration of cardiac and vascular structure [9]. It is known that cardiovascular structure and function differ between men and women. These differences in cardiovascular physiology are partly programmed by variation in male and female sex hormones [10]. For instance, stroke, hypertension, and atherosclerosis [11] are less common in healthy premenopausal women compared with age-matched men, but these differences disappear in the postmenopausal years [12]. Furthermore, the decline in sex hormones with aging adversely remodels the cardiovascular system in a sex-specific manner [13].

The purpose of this review is to highlight sex disparities in the cardiovascular system and the sex-specific impacts of exercise in cardiovascular remodeling from both animal and human studies. We summarize potential sex-specific mechanisms underlying cardiovascular remodeling, shed light on the gaps in knowledge in the field, and propose future studies to advance the field.

## 2. Sex Disparity in Cardiac Structure and Function

It has long been acknowledged that sex differences exist in cardiovascular structure and function after adjusting for determining factors, such as height, weight, and body composition. In general, women have smaller and lighter hearts, and likewise, their coronary arteries are also smaller than their male counterparts [14,15]. Sex difference in the cardiac structure becomes apparent at puberty, coinciding with the rise in sex hormones. In the pubertal age group, left ventricular (LV) mass is greater in boys than that in girls and chamber dimension, wall thickness, and myocyte hypertrophy likely contribute to the higher LV mass in males beyond puberty [16,17].

As age advances, the male heart loses on an average, one gram of cardiomyocytes daily, while the adequate heart mass is maintained. However, adult female hearts exhibit more resistance to cardiac cell loss and hypertrophy compared to their male counterparts [17,18,19]. Female sex hormones in general and estrogen in particular are thought to contribute to the prevention of cardiac hypertrophy in females. Several studies have demonstrated that estrogen attenuates cardiomyocyte apoptosis and modulates both physiological and pathological LV hypertrophy [20,21,22,23,24]. On the contrary, the loss of cardiomyocytes in males may be secondary to elevated levels of testosterone and/or epinephrine. These hormones have been associated with apoptosis and fibrosis [25,26,27].

Similarly, there are sex differences in cardiac function. For instance, the diastolic function is greater in premenopausal women compared to age-matched men [17,28], but this difference disappears in the postmenopausal years. Though the diastolic function decreases in both sexes with aging, reduction in systolic function occurs mainly in men [17,19]. Furthermore, studies show that the LV dimensions and functions also vary among men and women. Women have smaller LV chambers and reduced stroke volume (SV) compared to men [29]. Therefore, women rely on a higher heart rate compared to men to maintain similar cardiac output.

Sex differences are also seen in cardiac metabolism. Cardiac function is dependent on substrate oxidation (fatty acid, glucose, lactate, ketone, triglyceride, and glycogen) for the production of ATP [30]. Sex hormones may play a role in energy metabolism in the myocardium. For instance, a study performed in twenty-five young, healthy adults showed that women’s hearts use less glucose and more oxygen compared to the age-matched men [30]. Moreover, estrogen is known to increase endothelial NO synthase (eNOS), and its upregulation causes a decrease in translocation of glucose transporter (GLUT)-4 to the cell surface, thus inhibiting the glucose uptake and utilization in cardiomyocytes [11,31]. From these observations, it can be postulated that female hearts depend more on fatty-acid oxidation for myocardial energetics. Successively, they require more oxygen consumption, necessary for higher energy production, to meet the higher cardiac function compared to males [31]. Therefore, the sex-specific cardioprotection seen in females may only be limited to a well-perfused and oxygenated, healthy heart. In contrast, during cardiac stress and disease, the female myocardium may be less likely to adapt to make a shift in substrate metabolism.

## 3. Sex Disparity in Vascular Wall Structure and Function

There are several studies that show the protective role of estrogen in the vasculature, with fewer studies focusing on the impact of androgens on vasculature.

Male sex steroids have been linked to the enhancement of large artery stiffness [32]. During their reproductive years, female arteries exhibit lower arterial stiffness and prevalence of hypertension compared to age-matched males [33]. However, menopausal women exhibit stiffer large arteries than their male counterparts [32,33], manifesting in elevated prevalence of hypertension [34]. Berry et al. showed that elderly hypertensive women have stiffer large arteries, greater central wave reflection, and higher pulse pressure than elderly men [35]. Furthermore, in a longitudinal multiethnic cohort, Stern et al. reported that carotid arterial stiffening associated with aging could lead to an increase in systolic blood pressure in both sexes [36]; however, race and ethnicity (in men) and level of education (in women) could be further contributed to the differences between the sexes.

Many of estrogen’s beneficial vascular effects are related to modifying the functional state of the endothelium [37]. A number of studies indicate that estrogen promotes the release of endothelium-derived relaxing factors that confer resistance against atherosclerotic events. It was previously reported that the administration of estrogen to ovariectomized rats improves vascular function through its effects on vasodilators, such as nitric oxide (NO) [38,39,40]. These findings have been confirmed in both human [20,41,42] and animal [43] studies.

The sex differences in vascular tone and hypertension may be also related to differences in the production of or sensitivity to endothelium-derived contracting factors, such as endothelin-1 (ET-1). The lower blood pressure in female compared with male spontaneously hypertensive rats (SHR) has been attributed to the decreased level of ET-1 in females [44]. Several studies have reported that the contraction to phenylephrine, an alpha adrenergic agonist, is greater in the aorta of the male than in that of the female rats [45,46,47,48].

Overall, there are sex differences in cardiovascular structure, function, and metabolism. Furthermore, sex-specific differences exist in CVD. Exercise has been shown to have beneficial effects on the cardiovascular system [8,49]. Understanding of sex differences in cardiovascular system provides important mechanistic information for sex-specific impacts of exercise in healthy and pathological conditions. Next, we will highlight the sex-specific impacts of exercise with relation to various types of exercise regimens on cardiovascular remodeling.

## 4. Exercise-Induced Cardiovascular Remodeling and Functional Adaptation

Various types and intensities of exercise uniquely determine the outcomes on cardiovascular remodeling, so it is important to define the different categories of exercise in order to understand its impact on the cardiovascular system [9,50]. Exercise can be defined as a form of physical activity involving planned, structured, and repetitive movements, with the intention of maintaining or improving physical fitness [51]. Often, both intensity and duration are calculated based on the body mass index and age of men and women. Exercise intensity may be divided into three categories (low, moderate, and high intensity, see Table 1) based on the metabolic equivalent of task value (MET). These categories are as follows: low (<3 METs), moderate (3–5.9 METs), and high/vigorous (≥6 METs) [52]. Exercise intensity can also be quantified by the maximum oxygen consumption (VO_2max_) or the maximum heart rate (HR_max_) during the exercise. For low to moderate exercise, this would be defined as approximately 45–70% of VO_2max_ or 55–74% of HR_max_ respectively [53,54]. For high-intensity exercise, this would be defined as greater than 70% of VO_2max_ or greater than 90% of HR_max_ [53,54,55]. Of interest to this review, women exhibit a (5–15%) lower VO_2max_ compared with men when controlled for age and weight or activity [56]. A lower cardiac output is shown to contribute to a lower VO_2max_ in women in some studies [57,58]. Some pre-clinical and clinical studies have also characterized exercise training by endurance and strength training. It has been shown that various forms of exercise induce different types of EICR [59]. In this review, we will focus on exercise regimens including aerobic exercise (low–moderate and high-intensity) and combined exercise (aerobic and resistance training) (see Table 2).

In animal studies, unlike humans, exercise-training conditions are precisely defined and include forced treadmill training protocols, swimming, and cage wheel running [60]. Several animal studies have shown sex-specific differences in physiological hypertrophy with exercise. For instance, female rodents show greater physiological hypertrophy compared to male rodents with both types of swim and treadmill training [60,61,62,63]. Interestingly, female rodents can run faster and for greater duration compared to males when controlled for age and strain [63]. Furthermore, key metabolic pathways, including phosphoinositide-3 kinase (PI_3_K)/Protein Kinase B (AKT) pathway and calcium-calmodulin dependent kinase (CaMK) pathways, are impacted by exercise and associated with physiological hypertrophy in animal studies [8,49]. Exercised female groups also exhibit an increased phosphorylation of a negative inhibitor of physiological hypertrophy, Glycogen Synthase Kinase-3-beta (GSK-3-beta), an upstream mediator of AKT phosphorylation, that led to increased hypertrophic growth in the hearts of this group [63]. Furthermore, exercise selectively upregulates CaMK pathway in female rodents compared to exercised male rodents [63]. In contrast, sedentary male rodents exhibit selective upregulation of CaMK pathway compared to sedentary females [63].

Renin–angiotensin system (RAS) also plays an important role in the progression of cardiac remodeling. A decrease in the angiotensin-converting enzyme (ACE)/angiotensin (Ang) II/Ang II type 1 receptor (AT1) axis of RAS provides protection from pathological cardiac hypertrophy and subsequent heart failure [64,65]. Activation of AT1 receptor by Ang II has been shown to increase collagen and myocyte hypertrophy [64]. However, activation of angiotensin-converting enzyme 2 (ACE2) leads to the formation of Angiotensin (1–7)/Mas, which exhibits vasodilatory, anti-proliferative, and anti-trophic effects [66,67,68]. One study showed that six weeks of swimming training in FVB/N mice lacking Mas induced cardiac hypertrophy, which was associated to an increase in collagen I and III mRNA expression [66]. The investigators suggested that the increase in collagen attributed to an inversion of the balance between Ang II and Angiotensin (1–7) actions in the heart of Mas-knockout mice, favoring a stronger and unopposed influence of Ang II. Their data indicate that Angiotensin (1–7)/Mas axis is an important counter-regulatory mechanism in physical training-mediated cardiac adaptations [66]. 

In human studies, concentric thickening of LV is observed in strength training, whereas eccentric increase in cavity size of LV is more pronounced in endurance training [59,69]. There are structural and functional differences between the cardiovascular systems of young, trained athletes and the untrained ones. The LV cavity dimension and maximal wall thickness are greater in athletic females than the nonathletic females [70]. Moreover, the remodeling is different in males and females. The LV cavity dimension and wall thickness are smaller in female athletes compared to the male athletes of the same age, race, and sport disciplines [70]. Furthermore, women tend to develop eccentric hypertrophy, while concentric hypertrophy may be a normal finding in male athletes [71]. Women also exhibit improved endurance capacity and have increased catecholamine-induced fatty acid uptake and oxidation with exercise training compared to males [72,73]. Sex differences are also seen in SV with aging during submaximal and maximal exercise; older women have a lower SV and a smaller increase in SV from rest compared to aging men. This difference in SV is thought to in part be secondary to differences in LV remodeling with exercise and differences in lean body mass between the sexes [56]. In contrast, there is no difference in SV, cardiac index, LV end diastolic, and systolic volume indexes in younger (<40 years old) men and women [56].

Interestingly, exercise has variable impact on different heart chambers. For instance, intensive training-induced hemodynamic changes have been found to cause atrial enlargement in competitive athletes. A meta-analysis conducted by Iskandar et al. demonstrated that elite athletes have larger left atrium (LA) diameter and volume compared to control subjects [74]. The atrial enlargement, which is dynamic and reversible, is an adaptive mechanism in response to the increased training-induced volume overload, and it may vary by sex. In a study consisting of young elite rowers (46.5% women), LA enlargement was found more frequently in men than in women [75]. Similar to the LA, greater right atrium (RA) dimensions were observed in elite athletes compared to sedentary control subjects [76]. In addition, sex differences in EICR were also found in a study conducted in highly trained university athletes. While the male athletes showed LA and LV remodeling, right ventricular (RV) remodeling was significantly more common in female athletes [77]. Overall, the impact of exercise on sex-specific cardiac remodeling is greater in untrained men and women than in highly trained athletes [56]. 

Beneficial effects of exercise are also noted in the vascular endothelium [78]. For instance, the levels of endothelial-derived ET-1 and NO are impacted by exercise [79,80]. Exercise-induced increased cardiac output and intermittent increase in laminar shear stress contribute to vascular endothelial remodeling by activation of the PI_3_K/AKT pathway, leading to eNOS phosphorylation and subsequent increase in NO production [80]. NO-mediated endothelium-dependent vasorelaxation is also improved with exercise training [78]. NO is a key mediator of angiogenesis [80,81]. Up-regulation of vascular endothelial growth factor (VEGF) during exercise is required for angiogenesis associated with exercise [80]. However, one study in the literature reported both the basal levels of VEGF and response to exercise in the patients with cardiac failure was not different from the controls [82]. In a study of healthy male subjects, strength training led to an increase in ET-1, leading to increase in aortic pulse wave velocity (PWV), an established indicator of vascular stiffness. On the other hand, the same study showed that endurance training down regulated ET-1 and thereby reduced arterial stiffness [79]. Vascular endothelial dysfunction is a hallmark of the vascular disease; it is defined as a reduced endothelium-dependent vasodilation to vasodilators, such as acetylcholine (ACh) or flow-mediated dilation (FMD). Thus, the responses of vessels to ACh or FMD are used as a reproducible parameter to investigate endothelial function following exercise.

In a study of aging adults, twenty-four weeks of exercise led to improvement in vascular FMD and function in women but not in men [83]. In contrast, eight weeks of exercise led to the improvement of FMD in aging men but not women [84], suggesting that the duration of exercise may play a role in its sex-specific outcome. Another study in the literature demonstrated that exercise led to an increase in shear stress-mediated vasorelaxation in the brachial artery; however, this study did not include female subjects [85]. Overall, sex-specific impacts of exercise have been studied in the vasculature; however, data are sparse. Interestingly, beneficial roles of estrogen in combination with exercise are reported in postmenopausal women. In a study by Moreau et al., endurance training for twelve weeks in conjunction with estrogen treatment increased FMD in brachial arteries of postmenopausal women [86].

Beneficial impacts of exercise on cardiovascular structure and function are also seen in CVD, such as ischemia-reperfusion injury (IRI) and chronic heart failure (CHF). Endurance exercise has been reported to reduce oxidative stress and structural damage in IRI, thereby preventing myocardial dysfunction in animal studies [87,88,89,90,91,92]. However, the role of biological sex (if any) on the effects of exercise on myocardial oxidative stress is not clear. This is simply because the majority of studies did not include both sexes. Endurance training is also shown to reverse LV remodeling, with modest improvement in ejection fraction (EF) and LV end diastolic volume in CHF patients [93,94]. These studies were, however, performed only in males or the sex of the enrolled patients was not noted.

Animal studies provide further mechanistic insights into attenuation of adverse remodeling associated with heart failure. In a rodent model of dilated cardiomyopathy, swim training activated PI_3_K and improved survival. However, this investigation was performed only in males, and females were not studied [95]. Exercise has also been reported to upregulate sarco/endoplasmic reticulum Ca^2+^-ATPase (SERCA) pump in pathological hypertrophy, thus improving calcium handling and contraction in animal models [96,97]. Moreover, it is shown that CHF-induced increased sympathetic activity is reduced with exercise. For instance, eight weeks of exercise training reduced norepinephrine secretions by 16% [98]. Braith et al. demonstrated that endurance exercise reduced neuroendocrine activity by reducing the neuroendocrine hormones, including Ang II, vasopressin, and atrial natriuretic peptide; sex differences were not addressed [99]. Cardiac-failure-related increase in collagen deposition was also reduced in exercised animals; only males were studied [100].

Exercise has also shown to have beneficial effects on vascular diseases. Hambrecht et al. noted that four weeks of endurance exercise led to increase in phosphorylation of eNOS in the vessels of patients with coronary artery disease (CAD), thereby improving vasodilatory capacity [101]. Similarly, four weeks of exercise reduced Ang II in the vessels of male patients with CAD leading to reduced vasoconstriction; females were not studied [102].

## 5. Detrimental Impacts of Exercise

Despite the benefits of exercise on cardiovascular remodeling, there are also concerns that athletes are predisposed to the pathological cardiac remodeling, cardiovascular dysfunction, and arrhythmias.

### 5.1. Detrimental Impacts of Exercise on Cardiac Structure and Function

A meta-analysis reported that prolonged endurance exercise reduced RV function without causing any alteration to LV function in healthy individuals over 18 years of age [103]. A greater pulmonary artery pressure during exercise increases RV end-systolic wall stress, causing greater reduction in RV function [103]. Because exercise can impose a disproportionate physiological load on the RV compared to the LV, intense and prolonged exercise may result in long-term cardiac fatigue and remodeling [104,105,106]. A previous study suggests that some athletes suffer from serious arrhythmias emerging from pathological RV remodeling caused by chronic endurance exercise [107]. In fact, arrhythmogenic RV cardiomyopathy (ARVC) is an example of pathological remodeling and considered as one of the leading causes of sudden death in athletes [69,108,109,110]. However, since intense exercise may also lead to physiological adaptations of the heart, it could be difficult to differentiate between physiological RV remodeling and early-stage ARVC [69,110]. Cardiac magnetic resonance (CMR) has been utilized as a gold standard to assess RV structure and function and may help differentiate pathological remodeling from physiological remodeling [111].

### 5.2. Detrimental Impacts of Exercise on Cardiac Electrical Conductivity

Studies have shown an increased risk for developing atrial fibrillation (AF) in endurance athletes than in non-athletes [111,112]. Although data on the cardiac impact of endurance exercise in women athletes are scarce, studies have proposed that women are more protected from developing AF [112]. This sexual dimorphism could be explained by the difference in electrophysiological changes that occur in men and women during exercise. For instance, Wilhelm and co-workers observed significantly longer signal-averaged electrocardiogram (ECG) P-wave duration and increased incidence of AF in male cyclists compared to women cyclists [113]. The link between longer P-wave duration and increased risk of AF is already well known [114]. Animal studies have also corroborated with increased susceptibility to AF with chronic endurance exercise [115]. Moreover, underlying cardiac conductivity defects, like prolonged QTc and Brugada syndrome, can also lead to cardiac arrhythmias [116,117,118,119,120] and may have the potential to cause detrimental cardiac outcome with aerobic activities. It is also important to note that there may be sex differences in arrhythmogenic potentials secondary to underlying conditions, such as catecholaminergic polymorphic ventricular tachycardia (CPVT) associated with cardiac ryanodine receptor 2 (*RyR2*) mutations and other cardiac pathologies [116,117,118,119,120,121,122,123,124,125].

### 5.3. Detrimental Impacts of Exercise on Vascular Structure and Function

Although there have been studies that showcase the positive outcomes of exercise on vasculature, there are also reports on detrimental effects of high-intensity exercise [126]. For example, high-intensity exercise has been shown to decrease FMD in male participants [127,128]. However, there are also reports of increased FMD with high-intensity exercise in both sexes [56,129]. Overall, studies have shown that the alteration in FMD is dependent on exercise intensity [128,130]. In addition, there are reports on the exercise-induced increase in markers of endothelial dysfunction including, Von Willebrand factor, and thrombomodulin (in males) and microRNA-126 (in both sexes) [131,132]. Furthermore, a study by Boos et al. found evidence of exercise-induced vascular damage with an increase in circulating endothelial cells in men and women with CAD [133]. Contrasting effects of exercise on vascular function are further summarized in reports by Sapp and Hagberg, 2018, and Adams, 2018 [126,134]. Additional studies are needed to document the impact of exercise intensity on detrimental vascular outcomes.

## 6. Sex-Specific Impact of Low–Moderate Intensity Exercise on Cardiovascular Remodeling

Low to moderate-intensity exercise includes any activity that uses equal or less than 5.9 METs. In humans, exercises such as walking (up to 6 km/h), dancing (3.3 METs), or stationary cycling with light effort (at 100 Watts is equal to 5.5 METs) are considered as low–moderate intensity exercises [52,135]. However, in animal models, the measure of intensity is frequently based on minutes per day of activity. For instance, in rodents, ≤90 min/day of swimming or voluntary cage wheel running are considered low to moderate exercise [136]. Table 3 contains details of the low–moderate intensity training regimens and the studies discussed below.

### 6.1. Sex Disparity in Cardiac Structure and Function with Low–Moderate Exercise

In this section, we will discuss both animal and human studies addressing sex disparities in cardiac structural and functional remodeling with low–moderate intensity exercise.

Animal studies have shown that moderate-intensity training (MIT) induces physiological cardiac hypertrophy [54,137]. Asif et al., in a study only examining male rats, showed that MIT led to an increase in cardiac mass. This study also showed an age-specific impact on EICR by demonstrating that exercise led to increased LV mass in both adult and adolescent rats, but an increase in the number of cardiomyocytes was only seen in adolescents [137]. Apart from the induction of physiological hypertrophy, MIT can also reduce age-induced pathological cardiac hypertrophy. A study looking at only aged male rats exhibited a reduction in their pathological cardiac hypertrophy when they underwent MIT [138]. Furthermore, the same study showed that MIT training led to reduction in cardiac inflammation and cardiac fibrosis in aged male rats [138].

Molecular mechanisms underlying the beneficial effects of MIT on physiological cardiac remodeling have been also described. Dworatzek et al. demonstrated that mice undergoing MIT exhibited the activation of PI_3_K/AKT signaling pathway in the LV, thereby leading to cardiac hypertrophy. However, when compared to males, female mice exhibited greater cardiac hypertrophy following MIT [139]. Furthermore, in the same study, estrogen beta receptor knockout female mice had comparable cardiac structural change to the males, suggesting that sex differences observed in EICR are, in part, mediated via estrogen receptor signaling [139]. Beneficial effects of low–moderate exercise on cardiac function can be also mediated by other molecular mechanisms. For instance, rats undergoing MIT exhibited a decrease in collagen and an increase in citrate synthase levels in the LV [140]. Citrate synthase is necessary for aerobic capacity and mitochondrial mass, while increased collagen levels are associated with increased myocardial stiffness and heart failure [140,141]. Similarly, oxidative stress is known to contribute to pathological cardiac remodeling, and it is known that exercise training reduces oxidative stress in the heart [8].

Low to moderate-intensity exercise is shown to have several beneficial effects on cardiac function in animal studies. For instance, Hafstad et al. demonstrated that diet-induced obesity resulted in diastolic and systolic dysfunction in mice, and that could be prevented with MIT. However, females were not included in this study [142]. In another study, MIT resulted in decreased end systolic volume, increased SV, increased EF, and decreased LV pressure in male rats [140].

The effects of low–moderate intensity exercise on the cardiovascular system are not limited to animal models, as similar effects have also been demonstrated in human studies. In Dawes et al.’s study, moderate intensity, defined as exercise between three to five hours per week (assessed by a questionnaire), was associated with increased LV mass and increased LV and RV volume in healthy adult men and women; however, sex differences were not addressed, as patient data were grouped [143]. Another study found that in a community setting, intentional exercise, defined as the sum of activities that were consciously done for exercising, such as sports/dancing, conditioning activities, and walking regardless of the intensity level, was associated with increased LV mass, increased SV, and increased end diastolic volume in both men and women. Furthermore, the rate of increase in LV mass, SV, and end diastolic volume were more pronounced during lower intensity training in both sexes; however, the magnitude of changes was smaller in women than in men [9]. The underlying reason(s) for the sex differences in the beneficial impact of lower-intensity training in this study were unknown. However, the investigators made several speculations, including that the increased ventricular mass in men may be attributed to the larger hearts in males (despite accounting for baseline heart mass). Moreover, they suggested that the augmented changes in ventricular mass in men may have also involved participating in higher intensity of exercise compared to females [9].

### 6.2. Sex Disparity in Vascular Structure and Function with Low–Moderate Exercise

Similar to the beneficial impacts of low–moderate exercise on the heart, the beneficial impacts of exercise have been shown on vasculature. For instance, in a study only looking at male rats, Potora et al. demonstrated that that MIT resulted in hypertrophy and morphologic changes in aortic smooth muscle cells as well as an increase in the thickness of aortic elastic fibers. This increase in elastic fiber thickness was thought to lead to an increase in vascular distensibility, making the vessels better at handling mechanical stress [144].

Functional changes in the vasculature have also been observed with low–moderate intensity exercise. There has been increasing evidence suggesting a link between exercise and vascular ion channels [145,146,147]. Zhang et al. noted that aerobic exercise lowered systemic blood pressure and normalized hypertension-associated large-conductance calcium-activated potassium channels (BK_Ca_) upregulation to normotensive control levels in male SHR, and these effects were more pronounced in the moderate-intensity group than in the low-intensity group [148].

It has also been shown that low–moderate intensity exercise has beneficial effects on the peripheral vasculature in humans. One study in males demonstrated that moderate intensity exercise in the form of stationary cycling increases resting brachial artery diameter by 8%. Of note, brachial artery diameter is a measure of peripheral vessel function [149]. However, MIT did not result in changes in any of the other recorded cardiovascular measures, such as arterial stiffness, resting blood pressure, and heart rate; females were not studied, though [149]. The increase in brachial artery diameter noted with MIT is suggestive of structural remodeling that may be an adaptation to meet the blood-flow demands of exercise [149]. A similar study by Sawyer et al. also found that MIT resulted in increased brachial artery diameter. This study used both male and female subjects, but the investigators did not analyze them in separate groups; thus, no sex differences were elicited [150]. Another study that looked at the effects of high-volume endurance training (at 65% of VO_2max_) showed that popliteal artery distensibility and popliteal endothelial function were both improved [151]. However, no sex differences were observed in either popliteal artery distensibility or popliteal endothelial function with this mode of exercise [151]. In another study by Goto et al., MIT resulted in increased forearm blood flow in response to ACh through the increased production of NO. However, this study looked only at men [152]. Similarly, Sugawara and coworkers demonstrated that in post-menopausal women, both low and moderate-intensity exercise resulted in increased carotid arterial compliance. However, resting heart rate, blood pressure, and pulse pressure were not significantly changed in either low or moderate-intensity groups [153]. Furthermore, Sugawara and coworkers reported that both low and moderate-intensity exercise decreased low-density lipoprotein (LDL) but not high-density lipoprotein (HDL) cholesterol in post-menopausal women [153].

### 6.3. Limitations

The data detailing the impacts of low-moderate exercise on cardiovascular remodeling are limited. In particular, fewer studies address the effects of MIT in humans as compared to small animal models. Therefore, there is a need to translate some of the preclinical findings to humans and design prospective studies to address this gap. Another challenge in evaluating the effects of exercise on the cardiovascular system is the failure to uniformly define exercise intensity. In this review of the literature, we noted that some authors failed to report the VO_2max_ or METs used during training. Preclinical and clinical research should consider utilizing METs and VO_2 max_ to uniformly and accurately define low-moderate intensity exercise to assess its impact on cardiovascular remodeling. Furthermore, there is also need for more prospective studies on low-moderate exercise that include both male and female subjects and assess sex-specific impact of exercise on EICR. The sparse data currently available suggest that there are sex differences with low-moderate intensity exercise in cardiac remodeling; however, we are not able to ascertain similar differences in vascular remodeling [9,139].

## 7. Sex-Specific Impact of High-Intensity Training (HIT) on Cardiovascular Remodeling

HIT or vigorous exercise involves physical activity that requires ≥6 METs or greater than 70% of VO_2max_ or over 90% of maximal heart rate [52,53,54,55]. World Health Organization’s definition of vigorous exercise includes running, fast cycling, fast swimming, sports, or weightlifting that require greater effort and increased breathing and heart rate [154]. According to the U.S. Department of Health and Human Services 2018, 75–150 min/week of HIT is sufficient for substantial health benefit outcomes [155]. HIT has recently gained popularity in the form of high-intensity interval or intermittent training (HIIT). HIIT is defined as an exercise that combines relatively short to long bursts of intense exercise with periods of rest or lower intensity exercise [156,157]. Though the workout period is relatively short, studies showed that HIIT produces health benefits similar to or greater than MIT [157,158,159,160]. HIT is a well-known therapeutic intervention that results in physiological cardiovascular remodeling and has proven therapeutic benefits in pathological cardiovascular remodeling in both animal models and human subjects [129,140,156,161,162,163]. However, controversies exist, as both beneficial and detrimental effects on cardiovascular system have been reported with HIT. Table 4 contains details of the HIT regimens and the studies discussed below.

### 7.1. Sex Disparity in Cardiac Structure and Function with HIT

Cardiac structural and functional benefits have been noted in the literature with both HIT and HIIT. Preclinical studies are suggestive of the beneficial impacts of HIT on physiological cardiac remodeling in a healthy state. One study in the literature found that high-intensity swimming resulted in increased phosphorylation of AKT in the myocardium in both male and female rats but to a greater degree in females, thus leading to more pronounced LV hypertrophy in females [164]. HIT also increased SV and improved contractility and stroke work in both sexes; however, improvement in diastolic function was only seen in male rats [164]. Similarly, in a study of healthy male rats (females were not included), high-intensity treadmill running induced physiological LV hypertrophy. Moreover, improved cardiac performance (including enhanced EF, cardiac output, and volume), reduced myocardial collagen content, and increased cardiac capillary density were observed [140]. In addition, de Oliveira et al. demonstrated that, in a study of only male mice overfed a diet high in fat or fructose, HIIT reduced LV mass and LV wall thickness. These beneficial effects of HIIT were attributed to elevation in the components of the cardiac RAS, ACE2/Angiotensin (1–7)/Mas receptor [156]. As previously stated, activation of Angiotensin (1–7) in the myocardium is associated with vasodilation and antifibrotic, anti-hypertrophic, and antiarrhythmic actions [165,166]. RAS further modulates ACE2/Angiotensin (1–7)/Mas receptor axis, thereby exerting an anti-inflammatory effect in the myocardium, leading to beneficial impact on cardiac remodeling [167,168].

HIT has also been reported to have favorable effects in the disease states. Exclusively in a male rat model of pulmonary hypertension, high-intensity treadmill training enhanced RV apelin (a potent vasodilator) expression [169], leading to decreased RV systolic pressure, RV hypertrophy, fibrosis, and improved cardiac output [161]. HIIT training was also reported to have cardioprotective effects in male rats with IRI [170]. Rahimi and coworkers showed that the infarct size in the exercised male groups reduced by 50% and 35% after one and seven days post exercise, respectively, compared to the sedentary group [170]; females were not included. However, the beneficial effects of HIT on ischemic heart were lost after fourteen days following detraining. These investigators, therefore, proposed that the beneficial impacts of short-term HIIT may persist for a short period following exercise [170]. Of note, detraining is a state of decline in exercise-induced physiological conditioning due to insufficient training or pause in training.

In a study performed in healthy, middle-aged male individuals, HIIT induced beneficial RV hypertrophy, improved RV end systolic and end diastolic volumes, and decreased right ventricular ejection fraction (RVEF) and RV glucose uptake. However, RV mass, SV, and RV free fatty acid uptake remained unchanged in this cohort [171]. In another study that included both men and women participants, HIIT reversed pathological LV remodeling, reduced LV end-diastolic and end-systolic volumes, and improved left ventricular ejection fraction (LVEF) in stable post-infarction heart failure patients; sex differences were not addressed [55]. Furthermore, Wisløff and coworkers postulated that the beneficial impact of HIIT on post-infarction cardiac remodeling may have involved reduction in plasma pro-natriuretic peptide (B-type natriuretic peptide (BNP)) level, a marker of hypertrophy and severity of heart failure [55]. Beneficial cardiovascular effects of HIIT were also seen in aging men. In a study of sedentary aging men and aging male athletes that underwent aerobic preconditioning exercise or regular exercise regimen, respectively, this particular regimen of HIIT training resulted in improved resting blood pressure in both groups without the development of pathological cardiac remodeling [172]. The underlying mechanism(s) of improved cardiac structure and function with HIIT is not clear [156,162,173]. The beneficial effects of HIIT regimen may be attributed to the intermittent periods of rest that allows recovery, thereby enabling patients to complete the activity while also building their aerobic and anaerobic capacity [55].

Although HIIT is shown to have beneficial effects on cardiac structure and function, reduced ventricular function has also been reported with HIT. In healthy males, high-intensity endurance training caused a greater reduction in global RV strain, which was more pronounced in the RV free wall. Interestingly, a decrease in global LV strain was also observed, which was more pronounced in the LV septum [174]. This observation indicates that HIT may induce segment-specific cardiac dysfunction as well. Detrimental effects of HIT are also seen in the form of cardiac fatigue [8,175,176]. Data are not clear on sex differences in cardiac fatigue with HIT. Cote et al. noted that cardiac fatigue was more pronounced in males compared to females during a HIT (triathlon lasting six hours) [175]. However, sex differences were not noted in cardiac fatigue with prolonged HIT consisting of ultramarathon [175]. Further studies are warranted addressing the sex-specific detrimental effects of HIT.

### 7.2. Sex Disparity in Vascular Wall Structure and Function with HIT

The beneficial effects of HIIT are also reported on the vasculature. A study by Batacan Jr et al. reported that in adult male rats overfed with a high-fat high carbohydrate (HFHC) diet, HIIT decreased the contractile responses of mesenteric arteries to α-adrenergic stimuli and improved endothelium-dependent vasorelaxation to ACh [177]. These data were promising, as the investigators of this study demonstrated that HIIT was capable of alleviating mesenteric arterial contraction in these male rats despite being on HFHC diet. Females were not included in this study [177]. HIIT also improved vascular wall structure and function in human subjects. HIIT decreased systolic blood pressure in postmenopausal women; males were not studied [178]. In sedentary healthy adults (the sex of the subjects was not indicated), HIIT improved vascular function by increasing FMD of brachial arteries and decreasing aortic PWV, as indicators of endothelial function and arterial wall stiffness, respectively. These results suggest that HIIT alters vascular hemodynamic, thereby decreasing arterial wall thickness and enhancing endothelial function [129]. Similarly, improved FMD of brachial arteries and decreased oxidative stress were reported in the heart failure patients who underwent HIIT. The researchers postulated that the augmented NO bioavailability and plasma antioxidant level might have contributed to the beneficial impacts of HIIT on the vasculature of cardiac failure patients. Both males and females were included in this study, but the investigators did not analyze the sex differences in vascular remodeling [55]. In addition, Rognmo et al. [179] and Freyssin et al. [180] reported that HIIT intervention in both male and female patients with CAD and heart failure was safe and was not associated with adverse cardiac outcomes.

Overall, the data are sparse on the beneficial impacts of HIT on the vasculature, which has been mostly shown to have either detrimental or no effect on the vasculature. For instance, a clinical study conducted only in healthy males showed that HIT did not change the resting brachial artery diameter (a measure of peripheral vessel function) [149]. Furthermore, exclusively in male SHR, HIT adversely altered mesenteric arterial endothelial ultrastructure and function. In the same study, HIT increased oxidative stress and reduced NO bioavailability [181]. Similarly, Chen et al. reported that HIT worsened hypertension and intensified adverse remodeling of L-type voltage-gated Ca^2+^ (Ca_v_1.2) channels (upregulation of Ca_v_1.2 channels is a hallmark feature of hypertension) in mesenteric arteries of male SHR; females were not studied [182]. Investigators postulate that the detrimental effects of HIT may be reversed by incorporating HIIT since it exerts potentially greater effects compared to traditional endurance exercise [158]. Overall, HIIT has a more beneficial impact on the cardiovascular system compared to HIT.

### 7.3. Limitations

The sex-specific impacts of HIT and HIIT on cardiovascular remodeling is not well documented in either animal or human studies. Traditionally, the majority of studies on the impacts of HIT or HIIT have excluded females. Furthermore, those studies that did assess the impacts of HIT on cardiovascular remodeling using both male and female subjects did not attempt to address sex differences in their findings [55,162,179,180], thus preventing the assessment of sex-specific impacts of HIT or HIIT on cardiovascular remodeling. Therefore, the relatively small number of studies that addressed sex differences in the impacts of HIT or HIIT on cardiovascular remodeling limits us from drawing any significant conclusion. Though some studies have shown benefits of HIT on cardiovascular remodeling in CVD, there is a lack of HIT or HIIT studies in healthy subjects. Furthermore, variables, such as strain of animals, human race, age, fluctuations in sex hormones in menstruating females, menopause, diet, and varying HIIT programs (duration, intensity, and frequency) in different studies hamper our ability to assess the impacts of HIT or HIIT on the underlying mechanisms leading to cardiovascular remodeling. Moreover, differences between animal models and humans make it difficult to translate the findings in small-animal models to human studies.

## 8. Sex-Specific Impact of Combined Exercise on Cardiovascular Remodeling

Combined exercise trains more muscle groups at once in a relatively short period of time. Combined exercise has been demonstrated to offer more cardiovascular benefits in patients with CVD compared to any single exercise modality in several randomized control trials [183,184,185]. There are different types of combined exercise training, such as aerobic-resistance, aerobic-strength, and endurance-resistance, amongst others. The combination of aerobic and resistance training is frequently used as a combined form of exercise in humans. According to the American College of Sports Medicine (ACSM), aerobic exercise is any activity that increases the capacity of the cardiorespiratory system by increasing oxygen supply and improving the oxygen utilization in the muscles [186]. Examples of aerobic exercise include walking, swimming, dancing, cycling, jogging, and hiking [187]. Resistance training is a type of exercise that includes the use of load, machinery, or your own body weight while exercising the muscles [188]. Strength training and endurance training, however, are subtypes of resistance training. Strength training usually involves a load of 85% or more of one repetition maximum (1RM) for six or less repetitions of two to six sets with rest periods of two to five minutes. Muscular endurance training, on the other hand, requires resistance training at a load of 67% or less of 1RM for twelve or more repetitions of two to three sets with rest periods of thirty seconds or less [189]. We will discuss both animal and human studies addressing the sex disparities in cardiac structure and function with combined exercise below (please refer to Table 5 for details of the exercise regimens).

### 8.1. Sex Disparity in Cardiac Structure and Function with Combined Exercise

Combined exercise has been shown to improve adverse cardiac remodeling associated with hypertension and aging [190]. In a study conducted only in menopausal female SHR, aerobic-resistance training has been shown to improve cardiac function and decrease heart rates of experimental subjects [191]. Furthermore, Shimojo and coworkers showed that these improvements in the cardiac functions resulted from exercise-augmented cardiovascular autonomic modulation, decreased levels of tumor necrosis factor (TNF) and interleukin-6 (IL-6), reduced nicotinamide adenine dinucleotide phosphate (NADPH) oxidase (one of the major sources of superoxide in the cardiovascular system), and elevated levels of enzymatic or non-enzymatic antioxidants [191].

Clinical studies also concur with the beneficial effects of combined training on cardiac health. A randomized phase III clinical trial that included both male and female patients with heart failure showed that aerobic-strength training improved the LV diastolic function. However, this study did not analyze potential sex-specific impacts of combined exercise [192]. Similarly, in a randomized control trial of post-myocardial infarction (MI) male patients, combined aerobic resistance training showed improvements in LVEF and diastolic function. Females, however, were not included in this clinical trial [184]. Furthermore, Beckers and coworkers showed that combined endurance-resistance improved the LVEF in male and female patients with CHF, but sex differences were not addressed [183]. In contrast, a meta-analysis conducted in clinically stable male and female CHF patients noted that though aerobic training reversed LV remodeling, this favorable effect was not confirmed when aerobic training was combined with strength training [193]. In the above study, Haykowsky et al. reasoned that lack of improvement in cardiac function when aerobic exercise was combined with strength training could be due to the unfavorable outcome of strength training in cardiac failure patients [193]. Of note, strength training is shown to adversely alter cardiovascular function by increasing systolic and diastolic pressure loading and increasing LV wall stress [193].

### 8.2. Sex Disparity in Vascular Wall Structure and Function with Combined Exercise

Several studies have demonstrated the beneficial effects of combined exercise on vascular wall structure and function. One study found that combined aerobic-resistance training enhanced the expression of VEGF, an important angiogenic factor, in the skeletal muscle of the fifty-week-old obese male rats. This study did not include female subjects [194]. A study that was conducted exclusively in menopausal SHR showed that aerobic-resistance training decreased mean arterial blood pressure [191]. In this study, Shimojo et al. reported that exercise improved baroreflex sensitivity to increased blood pressure, leading to decreased sympathetic activation of peripheral vessels and reduced arterial blood pressure in those menopausal SHR [191]. Impaired baroreflex sensitivity is common and a strong predictor of arterial hypertension and cardiac mortality [195,196].

Beneficial effects of combined training on vascular function were also noted in human studies. Combined aerobic-resistance training decreased arterial stiffness and blood pressure in hypertensive postmenopausal women [197]. Son et al. reported that the decreased arterial stiffness in hypertensive postmenopausal women may have involved reduced ET-1 and increased NO (as measured by the level of nitrite/nitrate in blood) [197]. Thus, the potential underlying mechanisms for the combined training-mediated improvements in regulation of blood pressure in those subjects were attributed to the enhancement of vascular endothelial function or increased endothelium-dependent vasodilation [197,198]. However, the male subjects were not included in Son et al.’s study. In another study performed in premenopausal sedentary hypertensive women, aerobic-resistance training resulted in significant reduction in blood pressure and heart rate. The investigators of this study suggested that the reduction of vasomotor tone or increased vagal tone may have contributed to beneficial effects on blood pressure and heart rate that occurred after this exercise program [199]. Along the similar lines, Figueroa and coworkers reported that endurance-resistance training decreases blood pressure and arterial stiffness in postmenopausal women; men were not included in this study. These investigators attributed the beneficial effects of combined training on blood pressure to the enhanced endothelium-dependent vasodilation [200].

Studies in the literature showed that high-intensity resistance training might decrease arterial compliance in healthy men. Specifically, resistance training resulted in lowered carotid artery compliance in healthy young men [201]. In healthy middle-aged men (average years of resistance training was 21.3 ± 2.8 years), the carotid artery compliance was also decreased by 30% compared to their sedentary peers [202]. These studies did not include women. However, Kawano et al. demonstrated that resistance training in conjunction with aerobic training improves arterial compliance by decreasing arterial stiffness in healthy men (women were not included) [203]. These findings suggest that combined exercise may ameliorate vascular functions of men and women both in healthy and disease states. However, some controversies exist regarding the beneficial effects of combined exercise. In a previous meta-analysis, combined aerobic-resistance training and isolated aerobic training were reported to have nearly similar effects on arterial stiffness [204]. In contrast, a study performed in hypertensive older adults including both men and women demonstrated that aerobic-resistance training did not show any additional benefits in reducing blood pressure compared to resistance or aerobic training alone [205].

The order of exercise may play an important role on the outcomes of combined exercise. For instance, a study conducted only in older men [206] and another study done in healthy men and women [207] both showed that arterial stiffness was improved when aerobic exercise was performed after high-intensity resistance training, but no benefit was observed when the opposite order was followed. Another study on male and female normotensive and hypertensive adults also showed that aerobic exercise after resistance training exerted either no effects or some beneficial effects on arterial compliance [208]. Shiotsu et al. proposed that aerobic exercise after resistance training promoted arterial flexibility in older men by increasing the production of NO [206]. Moreover, Kawano et al. suggested that when aerobic exercise follows resistance training, it may prevent arterial stiffness associated with resistance training, but this benefit is lost when the exercise order is reversed [203]. None of above studies assessed the potential role of biological sex in relation to the order of exercise on the vascular structure or function.

### 8.3. Limitations

Sex differences in combined exercise on the cardiovascular remodeling have not been fully explored, as the majority of studies were performed either in males or females. Although some studies included both sexes, they did not isolate or compare the specific role of sex on the outcome of combined exercise. Furthermore, majority of studies were conducted in humans, with a relatively small sample size. Animal studies assessing the benefits of combined exercise on cardiovascular remodeling and functional adaptation are lacking. Moreover, the current literature has focused mainly on the impact of combined exercise on disease state rather than healthy state. With existence of various combinations of aerobic-strength training regimens, it is important to discern which combination is the most beneficial for cardiovascular health based on sex, age, and underlying conditions. Finally, although there are a number of studies that suggest beneficial roles of combined exercise, it should be noted that some studies reported no significant effects on the cardiovascular system [204,205].

## 9. Conclusion and Future Direction

Given that sex differences play an important role in cardiovascular physiology, the aim of this review was to highlight the studies in the literature that investigated sex-specific benefits of exercise on cardiovascular remodeling. The knowledge gained from current and future studies will ultimately (1) enhance our understanding of the mechanisms underlying the beneficial effects of exercise on cardiovascular function in healthy and disease states, (2) identify the role that biological sex may play in the impact of exercise on restoring cardiovascular function, and (3) provide insight into potential pathways for promoting healthy cardiovascular function and novel therapeutic targets for treating CVD.

Exercise training has emerged as a valuable modality in the prevention of CVD. Since cardiovascular physiology and CVD onset and progression vary between males and females, it is important to assess sex-specific roles of exercise training on the cardiovascular system. Throughout our review, we have underlined several (but not all) studies on the impacts of different types of exercise intensity and their relation to sex on the cardiovascular system. However, the data on sex differences in EICR are limited. Traditionally, studies on the effects of exercise have excluded females. Nevertheless, there has been an increasing number of investigations that have sought to include both sexes. On the other hand, regardless of several studies on EICR in males, there is still lack of consensus in the cardiovascular impacts of androgen in general. Androgens may simultaneously benefit and detriment the cardiovascular system by different mechanisms. Furthermore, most of the literature are focused on the outcomes of exercise in diseased models rather than healthy individuals. Therefore, future studies should be more inclusive of male and female subjects in both healthy and disease states. 

In the study of sex-specific impacts of EICR and functional adaptation, it is important to note that there are numerous physiological variables, including age, weight, race, and body composition (including fat and lean muscle mass), that play roles in EICR. Underlying genetic and molecular differences may also influence the structural remodeling pathways involved in cardiac hypertrophy, inflammation, fibrosis, and apoptosis. Moreover, the fluctuations in the levels of female sex hormone during different phases of the menstrual cycle may impact cardiovascular metabolism and function during exercise. Besides, a majority of the studies on the sex-specific impacts of EICR have not compared the effects of sex steroid hormones (e.g., estrogen vs. progesterone or testosterone vs. dihydrotestosterone (DHT)) on the cardiovascular system. In addition, the effects of female sex hormones signaling in cardiovascular physiology in men or the effects of androgen signaling in women are even less understood. Future investigations should examine the impacts of exercise on the cardiovascular system of the transgender population. 

Another possible factor that may impact cardiovascular remodeling is the form of exercise training undertaken; participation in varying types of exercise programs (modality, duration, intensity, and frequency). Future studies should utilize standardized, well-established exercise protocols to assess cardiovascular outcomes in both animal models and human subjects. They also need to assess the long-term effects of EICR and functional adaptation, as most of the studies only focus on short-term outcomes.

In conclusion, exercise has beneficial effects on the cardiovascular system, and it is an important component of health related to the immune system. To date, not many studies have directly addressed the sex-specific roles of different types of exercise on cardiovascular systems in both healthy and disease states. Therefore, it is essential to answer the following questions: (1) Are the cardiovascular protective effects of exercise sex specific? If so, what mechanisms are responsible for the sex-specific impacts of exercise on cardiovascular remodeling? And (2) does biological sex influence the outcome of EICR in disease states? 

**Figure 1:**
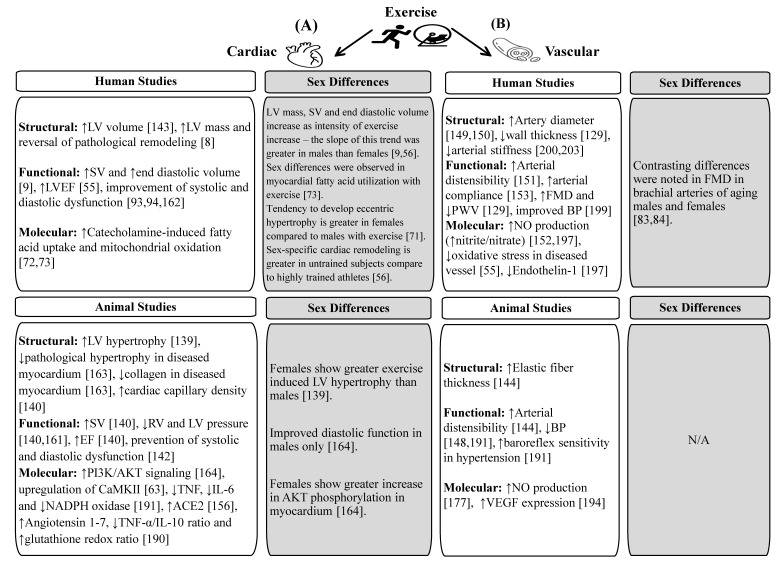
**Impacts of exercise and its sex-specific influences on cardiac (A) and vascular (B) remodeling in humans and animals.** ↓decrease, ↑increase, left ventricular (LV), stroke volume (SV), left ventricular ejection fraction (LVEF), flow-mediated dilation (FMD), pulse wave velocity (PWV), blood pressure (BP), nitric oxide (NO), right ventricular (RV), ejection fraction (EF), tumor necrosis factor (TNF), interleukin (IL), nicotinamide adenine dinucleotide phosphate (NADPH), angiotensin converting enzyme 2 (ACE2), vascular endothelial growth factor (VEGF).

Overall, the interest in more personalized approaches to the development of selective therapeutic strategies, including exercise, should further advance the studies of sex differences in cardiovascular physiology and pathophysiology. Figure 1 depicts the impacts of exercise and its sex-specific influences on cardiac and vascular remodeling in humans and animals based on studies presented in this review article.

## Figures and Tables

**Table 1:** Commonly accepted definitions of the different intensity levels of aerobic exercise.

Exercise Intensity	MET *	VO_2max_ *	HR_max_ *
Low	<3	<45%	<55%
Moderate	3–5.9	45–70%	55–74%
High	≥6	≥70%	≥90%

MET, metabolic equivalent of task; VO_2max_, maximum oxygen consumption; HR_max_, maximum heart rate. * Approximate ranges.

**Table 2:** Definitions of the different types of exercise.

Types of Exercise	
Aerobic	Activity that increases the capacity of the cardiorespiratory system by increasing oxygen supply and improving the oxygen utilization in muscles. Can be categorized further by intensity, such as low, moderate, and high.
Resistance	Exercise that includes the use of a load, machinery, or your own body weight to increase muscle strength and endurance. Strength and endurance training are subtypes of resistance training.
Combined	An exercise routine that incorporates a combination of aerobic and resistance exercise.

**Table 3:** The described studies associated with the low–moderate intensity training regimens.

Study	Participant Characteristics	Exercise Regimen	Cardiovascular Structural and Functional Findings	Sex-Specific Impact	Proposed Molecular Mechanisms
Asif et al. [137]	Male Wistar Kyoto rats	Running: treadmill Duration: up to 1 h/day, 5 days/week for 4 weeks	↑LV diameter	N/A	↓Cardiac microRNA-208b in LV (authors believe this change to be insignificant)
Liao et al. [54]	Aged male Sprague-Dawley rats	Swimming Duration: gradually increased from 20 to 60 min/day, 5 days/week for 12 weeks	↓Pathological cardiac hypertrophy	N/A	Down regulation of ERK1/2/JNK and NFATc3
Dworatzek et al. [139]	Male and female C57BL/6J mice	Running: voluntary cage wheel Duration: 8 weeks	↑LV mass	Greater cardiac hypertrophy in females compared to males	Activation of PI_3_K/AKT signaling pathway by upregulating AKT/mTOR signaling leading to cardiac hypertrophy
Verboven et al. [140]	Male Sprague-Dawley rats	Running: treadmill Duration: 1 h/day, 5 days/week for 13 weeks	↓End systolic volume, ↑SV, ↑EF, and ↓LV pressure	N/A	N/A
Hafstad et al. [142]	Male C57BL/6J mice	Running: treadmill Duration: ~120 min/day, 5 days/week for 10 weeks	Prevention of diet induced diastolic and systolic dysfunction	N/A	N/A
Potora et al. [144]	Male Wistar rats	Swimming Duration: 15 min/day for 14 days	Aortic smooth muscle cells hypertrophy and morphological changes, ↑thickness of elastic fibers	N/A	N/A
Zhang et al. [148]	Male SHR	Running: treadmill Duration: 60 min/day, 5 days/week for 8 weeks	↓Systemic BP	N/A	Correcting the hypertension-associated BK_Ca_ channel remodeling and suppressing the pathological adaptations of BK_Ca_ channels that result from high BP
Dawes et al. [143]	Males and females	MIT as defined by the Copenhagen City Heart Study Leisure Time Physical Activity Questionnaire	↑LV mass and ↑LV and RV volume in males and females	Data were not analyzed for sex differences	N/A
Turkbey et al. [9]	Males and females	MIT as defined by the MESA Typical Week Physical Activity Survey	↑LV mass, ↑SV, and ↑end diastolic volume in both sexes	Males showed a greater increase in LV mass, SV, and end diastolic volume as the levels of physical activity increased when compared to females	N/A
Shenouda et al. [149]	Healthy males	Stationary cycling Duration: 45 min/day for 12 weeks	↑Brachial artery FMD but no change in PWV	N/A	N/A
Sawyer et al. [150]	Healthy males	Stationary cycling Duration: 40 min/day, 3 days/week for 8 weeks	↑Brachial artery diameter but no significant change in FMD	N/A	N/A
Rakobowchuk et al. [151]	Males and females	Stationary cycling Duration: 40–60 min/day, 5 days/week for 6 weeks	↑Relative FMD and improved distensibility in popliteal artery	No sex differences were seen	N/A
Goto et al. [152]	Healthy males	Stationary cycling Duration: 30 min/day, 5–7 days/week for 12 weeks	↑Endothelium-dependent vasodilation	N/A	↑Production of NO
Sugawara et al. [153]	Post-menopausal females	Stationary cycling Duration: 3–5 days/week for 12 weeks	↑Arterial compliance and ↓ LDL	N/A	N/A

↓decrease, ↑increase, left ventricular (LV), stroke volume (SV), ejection fraction (EF), spontaneously hypertensive rats (SHR), blood pressure (BP), large-conductance Ca^2+^-activated K^+^ channel (BK_Ca_), moderate-intensity training (MIT), right ventricular (RV), flow-mediated dilation (FMD), pulse wave velocity (PWV), nitric oxide (NO), low-density lipoprotein (LDL).

**Table 4:** The described studies associated with the high-intensity training (HIT) regimens.

Study	Participant Characteristics	Exercise Regimen	Cardiovascular Structural and Functional Findings	Sex-Specific Impact	Proposed Molecular Mechanisms
Oláh et al. [164]	Healthy male and female Wistar rats	Swimming Duration: 200 min/day, 5 days/week for 12 weeks	↑SV and ↑contractility and stroke work in both sexes	More pronounced LV hypertrophy in females than males, ↑diastolic function only in males	↑Phosphorylation of AKT in the myocardium in both sexes but to a greater degree in females, thus leading to more pronounced LV hypertrophy in females
Verboven et al. [140]	Healthy male Sprague-Dawley rats	Running: Treadmill Duration: 10 bouts, 5 days/week for 13 weeks Speed: 18 m/min at 30° inclination	Beneficial LV hypertrophy, ↑EF, ↑cardiac output and volume, ↓myocardial collagen content, ↑cardiac capillary density	N/A	↑Cardiac metabolism due to increased oxygen supplied by enhanced capillary density and ↑citrate synthase and complex II enzyme activity (measure of mitochondrial mass)
de Oliveira Sá et al. [156]	Male C57BL/6 mice, overfed a diet high in fat or fructose	Running: Treadmill Duration: 3 days/week for 12 weeks Speed: 45 m/min	↓LV mass and LV wall thickness	N/A	Modulated components of the cardiac RAS, ACE2/Angiotensin (1–7)/Mas receptor axis
Brown et al. [161]	Male Sprague Dawley rats with pulmonary arterial hypertension	Running: Treadmill Duration: 30 min/day, 5 times/week for 6 weeks	↓RV systolic pressure, ↓RV hypertrophy, ↓fibrosis, ↑cardiac output	N/A	↑RV apelin expression
Rahimi et al. [170]	Male Wistar rats with IRI	Running: Treadmill Duration: 76–85 min/day, 5 consecutive days	↓Infarct size by 50% and 35% after 1 and 7 days post exercise	N/A	N/A
Batacan Jr et al. [177]	Wistar adult male rats overfed with a high-fat high carbohydrate (HFHC) diet	Running: Treadmill Duration: 4 bouts, 5 days/week for 12 weeks Speed: 50 m/min at 10% inclination	No significant difference for SBP or HR before and after exercise, ↑endothelium-dependent relaxation to acetylcholine, ↓contractile responses of mesenteric arteries to α-adrenergic stimuli	N/A	N/A
Fang et al. [181]	Male SHR	Running: Treadmill Duration: 60 min/day, 5 days/week for 8 weeks Speed: 26–28 m/min (~75–85% of the maximal aerobic velocity)	↑SBP and ↑DBP	N/A	↑Oxidative stress, ↓NO bioavailability
Chen et al. [182]	Male SHR	Running: Treadmill Duration: 60 min/day, 5 days/week for 8 weeks Speed: 26–28 m/min (~75–85% of the maximal aerobic velocity)	Worsened hypertension	N/A	↑Adverse remodeling of L-type voltage-gated Ca^2+^ (Cav1.2) channels
Heiskanen et al. [171]	Healthy, middle-aged males	Cycle ergometer Duration: 6 sessions in 2 weeks HIIT session: 4–6 × 30 s all-out cycling/4 min recovery	Beneficial RV hypertrophy, ↑RV end systolic and end diastolic volumes, ↓RVEF, ↓RV glucose uptake, but RV mass, SV, and RV free fatty acid uptake remained unchanged	N/A	N/A
Stewart et al. [174]	Recreationally active, healthy males who were training >5 h/week	Cycle ergometer Duration: only one 90 min exercise session at 110% of gas exchange threshold (GET)	More pronounced decrements in RV function, ↓LV function only to the sites of septal myocardium	N/A	N/A
Wisløff et al. [55]	Male and female heart failure patients	Walking: Treadmill Duration: 38 min/day, 3 times/week for 12 weeks	Reversed pathological LV remodeling, ↓LV end-diastolic and end-systolic volumes, ↑LVEF, ↑brachial artery FMD	Sex differences were not studied	↓Plasma pro-BNP level, ↑NO bioavailability, ↑Plasma antioxidant level
Grace et al. [172]	Aging male non-athletes and aging male athletes	Sprints, cycle ergometers Duration: Once/5 days for 6 weeks	↑Resting BP in both groups without causing pathological remodeling, ↑diastolic septal thickness, and ↓chamber diameter only in athletes	N/A	N/A
Klonizakis et al. [178]	Postmenopausal females	Cycling Duration: 10 × 1-min intervals at 100% of peak power output, 6 sessions in 2 weeks	↓SBP, no improvement in brachial artery FMD	N/A	N/A
Ramírez-Vélez et al. [129]	Healthy adults (study did not mention the sex of the subjects)	Fast walking and running: Treadmill Duration: 4 × 4 min intervals at 85–95% of HRR, 3 days/week for 12 weeks	↑Brachial artery FMD, ↓aortic PWV	N/A	N/A
Shenouda et al. [149]	Healthy males	Cycling sprints Duration: 3 × 20 s sprint interval training for 10 min for 12 weeks	No change in brachial artery diameter	N/A	N/A

↓decrease, ↑increase, stroke volume (SV), left ventricular (LV), ejection fraction (EF), renin–angiotensin system (RAS), right ventricular (RV), ischemia-reperfusion injury (IRI), systolic blood pressure (SBP), heart rate (HR), spontaneously hypertensive rats (SHR), right ventricular ejection fraction (RVEF), diastolic blood pressure (DBP), nitric oxide (NO), left ventricular ejection fraction (LVEF), flow-mediated dilation (FMD), pro brain natriuretic peptide (pro-BNP), blood pressure (BP), heart rate reserve (HRR), pulse wave velocity (PWV).

**Table 5:** The described studies associated with the combined exercise regimens.

Study	Participant Characteristics	Exercise Regimen	Cardiovascular Structural and Functional Findings	Sex-Specific Impact	Proposed Molecular Mechanism
Shimojo et al. [191]	Menopausal female SHR	Aerobic: Running on treadmill, 1 h/day at ∼50–60% of maximal running speed Resistance: Ladder climbing, 15 climbs/session at 1st–2nd week: 30–40%; 3rd–5th week: 40–50%; and 6th–8th week: 40–60% of the maximal load Duration: 5 days/week for 8 weeks	↓HR, ↓mean arterial BP, ↑baroreflex sensitivity	N/A	↓TNF and IL-6, ↓NADPH oxidase, ↑level of enzymatic or non-enzymatic antioxidants
Chrysohoou et al. [192]	Male and female heart failure patients	Aerobic: Cycle ergometers for 45 min/day Resistance: 4 exercises (knee extension, seated chest press, peck deck and lateral pull-down) with a fitness equipment Duration: 3 days/week for 12 weeks	↓PWV, ↑SBP, ↑LV diastolic function	Sex differences were not studied	N/A
Dor-Haim et al. [184]	Male MI patients	Aerobic-resistance: 20 min of treadmill walking, 15 min of cycling and 10 min of hand cycle paddling, total 45 min/day Duration: Twice/week for 12 weeks	↑LVEF, ↑diastolic function	N/A	N/A
Beckers et al. [183]	Male and female CHF patients	1st–2nd month: 10 min endurance, 40 min resistance 3rd–4th month: 16 min endurance, 30 min resistance 5th–6th month: 10, 12 and 15 min of endurance, the reminder of exercise session spent on resistance training Duration: 70 sessions in 6 months	↑LVEF	Sex differences were not studied	NT-proBNP levels remained unchanged
Son et al. [197]	Postmenopausal hypertensive females	Aerobic-resistance: Exercise intensity was increased gradually from 40% to 70% of HRR/4 weeks Duration: 3 times/week for 12 weeks	↓Brachial-ankle PWV, ↓BP	N/A	↓Endothelin-1, ↑ NO (as measured by the level of nitrite/nitrate in blood)
Masroor et al. [199]	Premenopausal hypertensive females	Aerobic: Running on treadmill for 20 min/day at 50–80% of HR_max_ Resistance: 3 sets of 10 repetitions of 5 exercises at an intensity of 50–80% of 1RM Duration: 5 days/week for 4 weeks	↓BP, ↓HR	N/A	N/A
Figueroa et al. [200]	Postmenopausal females	Endurance: Walking on treadmill for 20 min at 60% of HR_max_ Resistance: 12 repetitions for 9 exercises for 20 min at 60% of HR_max_ Duration: 3 times/week for 12 weeks	↓Brachial-ankle PWV, ↓SBP and DBP, ↓HR	N/A	N/A
Kawano et al. [203]	Healthy males	Aerobic: Cycling for 30 min at 60% of HR_max_ Resistance: 3 sets of 8–12 exercises at 80% of 1RM Duration: 3 sessions/week for 4 months	↑Arterial compliance	N/A	N/A
Lima et al. [205]	Hypertensive older males and females	Aerobic: Treadmill ergometer, 1st–4th week for 25 min, 5th–10th week for 35 min Resistance: 1st–4th week: 9 exercises, 5th–10th week: 15 repetitions for the upper limbs and 20 repetitions for the trunk and lower limbs at 50 to 60% 1RM Duration: 3 training sessions/week for 10 weeks	Did not show any additional benefits in reducing BP compared to resistance or aerobic training alone	Sex differences were not studied	N/A
Shiotsu et al. [206]	Older males	Aerobic: Cycling for 20 min at 60% of HRR Resistance: 3 sets of 8–12 repetitions for 5 different exercises at 70–80% of 1RM Duration: Twice/week for 10 weeks	↓Carotid-femoral PWV	N/A	N/A
Okamoto et al. [207]	Healthy males and females	Aerobic: Running for 20 min at 60% of the targeted HR Resistance: 5 sets of 8–10 repetitions at 80% of 1RM, Duration: Twice/week for 8 weeks	↓Brachial-ankle PWV, ↑brachial artery FMD	Sex differences were not studied	N/A

↓decrease, ↑increase, spontaneously hypertensive rats (SHR), heart rate (HR), blood pressure (BP), tumor necrosis factor (TNF), interleukin-6 (IL-6), nicotinamide adenine dinucleotide phosphate (NADPH), pulse wave velocity (PWV), systolic blood pressure (SBP), left ventricular (LV), myocardial infarction (MI), left ventricular ejection fraction (LVEF), chronic heart failure (CHF), N-terminal pro b-type natriuretic peptide (NT-proBNP), nitric oxide (NO), maximum heart rate (HR_max_), diastolic blood pressure (DBP), heart rate reserve (HRR), 1 repetition maximum (1RM), flow-mediated dilation (FMD).
